# TGF-β: The Molecular Mechanisms of Atherosclerosis - insights into SMAD Pathways and Gene Therapy Prospects

**DOI:** 10.2174/0109298673373967250313053208

**Published:** 2025-04-15

**Authors:** Klaudia Bonowicz, Dominika Jerka, Karolina Ławkowska, Jolanta Łuniewska-Bury, Irena Wrońska, Yidong Bai, Maciej Gagat

**Affiliations:** 1 Department of Histology and Embryology, Collegium Medicum in Bydgoszcz, Nicolaus Copernicus University in Torun, 85-092, Bydgoszcz, Poland;; 2 Faculty of Medicine, Collegium Medicum, Mazovian Academy in Płock, 09-402, Płock, Poland;; 3 Department of Cell Systems and Anatomy, UT Health, Long School of Medicine, The University of Texas Health Science Center at San Antonio, San Antonio, TX 78229, USA

**Keywords:** Atherosclerosis, TGF-β, SMAD pathways, gene editing, gene loci, atheroma plaques

## Abstract

Atherosclerosis, a leading cause of global morbidity and mortality, is characterized by plaque formation resulting from the accumulation of fibrous elements, lipids, and calcification in arteries, leading to complications such as ischemic stroke, coronary artery disease, and myocardial infarction. Traditional treatments primarily address symptoms but fail to target underlying causes, prompting exploration of novel approaches like gene therapy. The TGF-β family, encompassing TGF-β1, TGF-β2, and TGF-β3, plays a critical role in cellular processes including proliferation, apoptosis, and migration, with its dysregulation strongly linked to cardiovascular diseases. In atherosclerosis, TGF-β influences key factors, such as macrophage cholesterol regulation, plaque stability, and vascular smooth muscle cell function, while also contributing to endothelial dysfunction-an early stage in disease development. Personalized medicine has highlighted the importance of tailoring therapies to genetic profiles, particularly regarding TGF-β pathway variations such as SNPs in TGF-β1 and TGFBR2, which could inform more precise interventions. Emerging technologies like CRISPR-Cas9 and RNA-based therapies enable targeted modulation of these genetic factors, offering new avenues to mitigate disease progression. CRISPR-Cas9 allows direct editing of gene loci linked to atherosclerosis, potentially correcting mutations or modulating expression levels, while RNA-based therapies, including siRNAs and antisense oligonucleotides, provide additional precision tools for addressing dysregulated genes. This review focuses on identifying key genes and additional molecular players involved in or regulated by the TGF-β pathway that may serve as precise targets for gene therapy intervention in atherosclerosis and related cardiovascular diseases. By targeting genes involved in cholesterol metabolism, inflammation, and endothelial function, gene therapy offers a targeted strategy to ameliorate the genetic drivers of these conditions. In summary, modulation of TGF-β signaling by gene therapy has the potential to revolutionize the treatment of atherosclerosis and other cardiovascular diseases while shedding light on the underlying genetic mechanisms of these disorders.

## 1 INTRODUCTION

Atherosclerosis, one of the foremost causes of illness and mortality globally, is a complex condition characterized by the accumulation of fibrous elements, lipids, and calcification within medium and large arteries. This disease initiates with endothelial activation and progresses through inflammatory pathways, leading to the formation of atheroma plaques [1]. Complications include ischemic stroke, coronary artery disease, and myocardial infarction. Traditional treatments like statins and antiplatelet drugs have drawbacks like liver damage and bleeding risk [2]. Despite significant research advancement over the past three decades, questions remain regarding the origins of atherosclerosis, prompting the exploration of new animal models and therapies. Understanding the mechanisms underlying atherosclerosis, from endothelial dysfunction to plaque rupture, is imperative for developing effective prevention and treatment strategies [1, 3, 4]. Atherosclerosis, with its genetic basis, is a potential target for gene therapy, which offers a novel approach by targeting specific genes involved in disease progression [4]. It aims to modulate key cellular processes, such as inflammation and plaque stability. This approach has the potential to halt disease progression and reduce the risk of acute cardiovascular events. Gene therapy represents a promising alternative to addressing the complexities of atherosclerosis [5].

Obstacles to the clinical application of gene therapy in cardiovascular diseases primarily include factors such as insufficient understanding of the nature of cardiovascular diseases, limitations of genetic technology, or ethical considerations. While the initiating factors are well-known, understanding the disease's relentless progression post-establishment remains a challenge [6]. Chronic vascular inflammation and abnormal mechanotransduction are thought to play a role in atherosclerosis progression, but their exact influence remains uncertain. Recent research indicates that activation of endothelial TGF-β signaling could be a driver of atherogenesis. TGF-β has been extensively studied in cardiovascular diseases, revealing its dual role in both promoting and protecting against pathogenesis. Given its wide-ranging effects, genes targeted by TGF-β/Smad signaling present intriguing therapeutic targets. Identifying and modulating the expression of these genes could be a beneficial strategy for therapy [7].

It is worth noting that the TGF-β signaling pathway has been widely studied in the literature for many years. However, conflicting results often arise in research findings. Despite these discrepancies, the broad and abundant nature of TGF-β, particularly within the artery wall, makes it crucial to systematically organize our knowledge of its role in atherosclerosis [8-10]. The dual role of TGF-β in cancer progression has been relatively well-explained in the literature, with its ability to either promote or inhibit tumor growth depending on the context [11]. Similarly, understanding its dual function in atherosclerosis could provide valuable insights into potential therapeutic strategies. Novel therapeutics that target TGF-β production or block its action are currently in pre-clinical or early clinical trials for cancer treatment and have shown promising results. These therapies primarily focus on inhibiting TGF-β-mediated SMAD signaling, which plays a pivotal role in disease progression. Further clinical trials are expected to define more precise drugs targeting TGF-β activity, contributing to improved outcomes in cancer therapies [12]. Given its widespread involvement in various diseases, the TGF-β pathway has also emerged as a target in other pathological conditions, including fibrotic and inflammatory diseases. Strategies aimed at preventing fibrosis, for instance, focus on modulating TGF-β signaling to reduce excessive tissue scarring and inflammation [13, 14]. Moreover, therapies targeting different TGF-β isoforms have shown enhanced efficacy and safety profiles, highlighting the importance of isoform-specific approaches in developing more effective treatments [15]. By expanding our understanding of how TGF-β contributes to processes contributing to atherosclerosis development, we can uncover potential therapeutic targets, helping to clarify its dual role and maximize its potential for clinical applications. Identifying and modulating the expression of these genes could be a prudent strategy for therapy.

## 2 TGF-β FAMILY AND SIGNALING PATHWAYS IN THE VASCULAR SYSTEM

### 2.1 TGF-β and the TGF-β Family

The transforming growth factor beta (TGFβ) family comprises a vast array of over 40 proteins, including TGF-β, inhibin, activin, growth differentiation factors (such as GDF-15), bone morphogenetic proteins (BMPs), nodal, and Mullerian-inhibiting substance. TGF-β1 serves as the prototype of this family, alongside closely related TGF-β2 and -β3, as well as more distantly related proteins such as activins, inhibins, nodal proteins, and BMPs. Their regulatory effects on cellular processes span various cell types and contexts, encompassing induction of proliferation, apoptosis, migration, adhesion, ECM protein production, and cytoskeletal organization [16]. Crucially, TGF-β family cytokines play pivotal roles in embryonic development, cell fate determination, tissue homeostasis, and repair. Dysregulation of TGF-β signaling can precipitate pathological conditions, including cardiovascular diseases, fibrotic disorders, and cancer, thus fueling intensive research into therapeutic interventions aimed at restoring balanced TGF-β signaling [17].

### 2.2 Route from Receptor to Gene Expression

The multifunctional nature of TGFβ signaling requires tight regulation at various levels, including ligand production, release from binding proteins, receptor activation, and transcriptional complex formation. These steps ensure timely activation and termination of TGFβ signaling [18]. The biosynthesis of TGF-β begins in the endoplasmic reticulum, where the precursor polypeptide undergoes cleavage to produce the mature cytokine and latency-associated peptide (LAP). This complex forms a small latent complex (SLC) that is either secreted as a large latent complex (LLC) by binding to latent TGF-β-binding proteins (LTBPs) or remains tethered to the cell surface through glycoprotein-A repetitions predominant protein (GARP) or leucine-rich repeat-containing protein 33 (LRRC33) [19, 20]. The activation of TGF-β from its latent state is a critical step in its regulation. Integrin-mediated activation is the most described mechanism, with αVβ6 and αVβ8 integrins playing central roles. These integrins bind to the RGD sequence in LAP, and through interactions with the actin cytoskeleton, they apply mechanical force to expose the receptor-binding domain of TGF-β, thus activating it [21, 22].

TGFβ signaling begins when a TGFβ ligand binds to serine/threonine kinase receptors on the cell surface, specifically, TGFβR1 and TGFβR2, which are the principal receptors in the vasculature [23]. TGFβR2, located on chromosome 3, and TGFβR1, located on chromosome 9, form heterodimer complexes to bind various TGFβ ligands and initiate signaling, with type III receptors like endoglin and betaglycan enhancing specificity and regulation by binding TGFβ isoforms and facilitating their interaction with TGFβR2. The binding of active, dimeric TGF-β triggers the phosphorylation of type I receptors by the constitutively active type II receptor, initiating a cascade of downstream signaling events. Central to TGF-β superfamily signaling are small mothers against decapentaplegic (Smad) transcription factors, which serve as the principal intracellular mediators. Among the eight Smad proteins expressed in mammals, Smads 2 and 3 function as the primary receptor-regulated Smads (R-Smads), activated by receptors for various TGF-β ligands. Additionally, Smad4, known as Co-Smad, acts as a universal partner for all R-Smads, facilitating their nuclear translocation and subsequent regulation of gene expression. Within the nucleus, R-Smad/Smad4 complexes directly bind to Smad-binding elements (SBE) in the promoters of TGF-β target genes. This interaction, coupled with the involvement of DNA binding co-factors, co-activators, and co-repressors, orchestrates precise gene expression response [24]. Such coordinated actions of Smad proteins and their transcriptional partners play a crucial role in regulating cell lineage-specific gene expression, thus contributing significantly to the modulation of endothelial and smooth muscle cell phenotype and function, particularly in the context of vascular homeostasis and disease [25].

Phosphorylation and dephosphorylation represent pivotal post-translational modifications (PTMs) essential for the regulation of TGF-β signaling. The activation of Smad2 and Smad3, key receptor-regulated Smads (R-Smads), occurs through the phosphorylation of two serine residues within their C-terminal SSXS motif by TGF-βRI [26]. This phosphorylation event is central to the canonical TGF-β signaling pathway. Beyond this, various kinases, notably from the mitogen-activated protein kinase (MAPK) family—such as p38 MAPK, c-Jun N-terminal kinase (JNK), and extracellular signal-regulated kinase (ERK)—phosphorylate specific sites within the linker regions of Smad2 and Smad3 [22]. These modifications influence their transcriptional activity, adjusting the signaling output in contexts like cardiovascular disease. Notably, the phosphorylation of Smad3 by MAPKs enhances its ability to drive the expression of pro-fibrotic genes such as CTGF and collagen I, both implicated in vascular remodeling processes [27].

Dephosphorylation plays an equally critical role in controlling TGF-β signaling. Protein phosphatases such as protein phosphatase magnesium-dependent 1A (PPM1A) catalyze the removal of phosphate groups from the C-terminal SSXS motif of Smad2 and Smad3, promoting their nuclear export and terminating the signaling process. A decrease in PPM1A levels has been associated with sustained phosphorylation of Smad3, leading to enhanced fibrotic responses in multiple disease states, including cardiovascular disorders. Furthermore, the recruitment of T cell protein tyrosine phosphatase (TCPTP) to TGF-βRII facilitates the dephosphorylation of tyrosine residues in the receptor's cytoplasmic tail, thereby attenuating fibrotic signaling [28].

Another essential regulatory layer within the TGF-β pathway involves ubiquitination and deubiquitination. Ubiquitination is primarily mediated by the ubiquitin-proteasome system (UPS), which governs the degradation of intracellular proteins, including Smads and TGF-β receptors. E3 ubiquitin ligases, such as SMAD ubiquitination regulatory factors (SMURFs), target R-Smads and inhibitory Smads (I-SMADs) like Smad7 for proteasomal degradation, thereby modulating the signaling cascade. Smurf2, specifically, ubiquitinates Smad2 and Smad3, facilitating their degradation and serving as a negative regulator of TGF-β signaling. Additionally, the E3 ligase Arkadia (RNF111) promotes TGF-β signaling by targeting Smad7 for degradation, effectively reducing the inhibitory impact of I-SMADs on the pathway [28]. Deubiquitination counteracts the process of ubiquitination and stabilizes critical components of the TGF-β signaling pathway. Deubiquitinating enzymes (DUBs) play a key role in this process by removing ubiquitin molecules from substrates [29]. For instance, USP15 has been shown to deubiquitinate and stabilize TGF-β receptors, thereby supporting sustained signal transmission. The balance between ubiquitination and deubiquitination is crucial for maintaining the appropriate dynamics of TGF-β signaling. Any imbalance in these processes can contribute to pathological outcomes, such as fibrosis and vascular inflammation, which are central features of cardiovascular diseases [30].

In recent years, increasing emphasis has been placed on the role of reactive oxygen species (ROS) as a key activator of TGF-β signaling pathways. Among the three isoforms, TGF-β1 stands out as the only one that can be directly activated by ROS, owing to the presence of a unique methionine residue at position 253 within its LAP, which undergoes an oxidation-induced conformational change. Nevertheless, ROS can also promote the activation of all TGF-β isoforms indirectly by inducing other activators such as thrombospondin-1 (TSP-1) and matrix metalloproteinases (MMPs). ROS-driven TGF-β activation is particularly prevalent in tissues exposed to asbestos, ultraviolet light, and ionizing radiation. Additionally, elevated glucose levels can enhance ROS production, leading to increased TGF-β activation, which contributes to the progression of fibrotic and inflammatory diseases [31, 32].

TGF-β signaling extends beyond its canonical Smad-dependent pathway through various non-canonical routes involving kinases like ERK, JNK, p38 MAPK, and components of the PI3K/AKT axis [33]. These non-canonical pathways play significant roles in cardiovascular diseases by regulating cell proliferation, apoptosis, and migration. For example, ERK activation by TGF-β has been linked to smooth muscle cell proliferation, a process contributing to neointimal hyperplasia and restenosis. Similarly, the PI3K/AKT pathway enhances cell survival and promotes ECM production, which is vital for the progression of atherosclerotic plaques [34].

## 3 QUEST FOR TGF-BETA'S ROLE IN ATHEROSCLEROSIS

In the exploration of TGF-β's role in atherosclerosis, one encounters a myriad of assertions regarding its function. Primarily, TGF-β exhibits expression and interacts with nearly all cells involved in the atherosclerotic process. These interactions encompass TGF-β's expression in vascular smooth muscle cells, endothelial cells, macrophages, platelets, and hematopoietic cells and its responsive engagement with signaling pathways implicated in inflammation, cell proliferation, and extracellular matrix remodeling [18]. Within the literature, TGF-β's properties are depicted as dual-fold: it is described as both anti-atherogenic, preventing atherosclerosis, and pro-atherosclerotic, stimulating its progression. TGF-β's anti-atherogenic attributes arise from its capacity to regulate genes associated with macrophage cholesterol levels. It initiates the activation of Smad-2 and -3 proteins, which oversee the expression of genes involved in both the uptake and efflux of cholesterol in macrophages. Specifically, TGF-β impedes the uptake of modified LDL through a Smad-dependent mechanism, with Smad-2 playing a pivotal role in gene expression regulation [35]. However, a study by Ahmadi *et al.* delved into the role of platelet-borne TGF-β1 in coronary artery disease (CAD), where activated platelets are implicated in atherosclerosis and atherothrombosis, culminating in CAD. Findings unveiled elevated levels of TGF-β1 in CAD patients, along with conspicuous bands of mature TGF-β1 in their platelets. Noteworthy correlations emerged between active TGF-β1 and pro-inflammatory markers in CAD patients. These findings suggest a notable involvement of platelet-borne TGF-β1 in CAD pathogenesis, accentuating its role in the progression of atherosclerosis [36]. TGF-β further demonstrates significant expression in cells composing the arterial wall, playing a pivotal role in regulating endothelial and vascular smooth muscle cell function, which is also elaborated on in Chapters 4 and 5.

The three main isoforms associated with atherosclerosis are TGF-β1, TGF-β2, and TGF-β3. The most extensively researched isoform of TGF-β in the context of atherosclerosis is TGF-β1. However, the current research addresses a significant gap in the existing literature by focusing on the roles of TGF-β2 and TGF-β3 in atherosclerosis. TGF-β1 is known for its potent anti-inflammatory, immunosuppressive effects within the immune system. However, it has also been associated with inflammation and autoimmunity when combined with specific cytokines and T cells [37]. A study by Andreas Edsfeldt and colleagues revealed the pivotal role of TGF-β2 in maintaining plaque stability. Through their analyses, the researchers identified TGF-β2 as a differentiating factor between asymptomatic and symptomatic plaques, underscoring its significance in plaque pathophysiology. Additionally, their findings suggest that TGF-β2 plays a role in plaque stabilization by enhancing collagen accumulation and inhibiting the matrix-degrading enzyme MMP-9, thereby mitigating the risk of plaque rupture [38]. Though there is limited information available regarding the role of TGF-β3 in atherosclerosis, its presence in atherosclerotic tissues suggests that it may also contribute to the pathogenesis of the disease. The latest research focuses on elucidating the mechanisms through which TGF-β3 regulates inflammatory reactions and facilitates the wound healing process. However, the results are inconclusive and underscore, as exemplified in the case of the entire TGF-β family, the pleiotropic nature of this isoform, elucidating the diverse roles of TGF-β3 across different phases of wound healing [39].

## 4 ROLE OF TGF-Β IN ENDOTHELIAL DYSFUNCTION AND ATHEROSCLEROSIS

Endothelial dysfunction marks an early stage in the onset of atherosclerosis, characterized by heightened levels of adhesion molecules and pro-inflammatory cytokines that disrupt normal endothelium-dependent vasodilation. As the primary regulator of vascular wall stability, the endothelium manages vascular permeability, and the adhesion and aggregation of platelets and leukocytes, as well as thrombosis. This dysfunction, driven by increased pro-inflammatory and pro-thrombotic factors, leads to impaired vasodilation. Evidence suggests that endothelial dysfunction not only initiates atherogenesis but also contributes to the development and complications of atherosclerotic plaques [40].

A phenomenon worth mentioning alongside endothelial dysfunction in atherosclerosis progression is endothelial-to-mesenchymal transition (EndMT). EndMT occurs when endothelial cells transform into mesenchymal stem cells, particularly under conditions of heightened oxidative stress, disturbed metabolism, hypoxia, and shear stress forces [41]. If a substantial proportion of endothelial cells experience EndMT, it can lead to disruptions in the endothelial layer, ultimately accelerating plaque erosion and exacerbating the advancement of atherosclerotic disease. Among the various signaling pathways implicated in EndMT, the TGF-β pathway stands out as particularly influential, especially its canonical downstream Smad pathway. Additionally, the crosstalk between the TGF-β pathway and Wnt/β-catenin signaling plays a regulatory role in EndMT. Furthermore, within the complex milieu of inflammation and oxidative stress, EndMT regulation becomes even more intricate. Activation of NF-κB can trigger EndMT, as evidenced by induction through IL-1β plus TGF-β2 or TNF-α and IL-6 stimulation of endothelial cells [42]. Numerous studies have confirmed the pivotal role of TGF-β in inducing EndMT. The three main isoforms of TGF-β (TGF-β1, TGF-β2, and TGF-β3) vary in their potency to induce EndMT, with TGF-β2 often acting as a key paracrine signal. Both canonical (Smad-dependent) and noncanonical (Smad-independent) TGF-β signaling pathways contribute to EndMT. In the canonical pathway, TGF-β activates its receptor complex (TGF-βR1/TGF-βR2), leading to phosphorylation of Smad proteins, particularly Smad2 and Smad3, which then associate with Smad4 and translocate to the nucleus to regulate the expression of genes involved in EndMT, such as Snai1, Snai2, and Twist1. Beyond the Smad-dependent pathway, noncanonical pathways involving kinases like Ras, Tak1, PI3K, and c-Abl provide alternative routes for TGF-β-induced EndMT [43]. A recent study reveals a metabolic shift in endothelial cells, driven by increased acetate production from glucose, as the foundation of TGF-β-induced EndMT [44]. These findings highlight the role of metabolic regulation in EndMT initiation and progression [44]. EndMT’s role in driving atherosclerosis forward makes it a potential target for anti-atherosclerosis treatments. Accordingly, hydrogen sulfide (H_2_S) has been found to have protective effects on the cardiovascular system due to its ability to reduce inflammation, oxidative stress, foam cell formation, regulate ion channels, and improve cell adhesion and endothelial function. However, the exact mechanism by which H_2_S works against atherosclerosis and its impact on EndMT is still unclear [45].

The human genome encodes a variety of type I and type II receptors for the TGF-β family, facilitating diverse ligand binding and intracellular signaling pathways. This diversity in receptor composition is pivotal in determining the cellular response to TGF-β. Specifically, TGF-β exhibits dual effects on endothelial cell proliferation, with its impact contingent upon the specific receptor complexes engaged. While TGF-β typically impedes endothelial cell proliferation *in vitro*, it is indispensable for vasculogenesis and angiogenesis *in vivo*, eliciting an angiogenic response. Endothelial cells express two principal types of TGF-β type I receptors: ALK-1 and ALK-5 (TβRI), each activating distinct Smad pathways [46]. Smad proteins are key mediators of the signaling pathway initiated by TGF-β. They regulate gene expression in response to TGF-β signaling, controlling processes such as cellular proliferation and extracellular matrix turnover. This pathway is crucial in vascular biology and is implicated in various cardiovascular diseases [47]. Activation of ALK-1 promotes endothelial cell proliferation and migration, whereas ALK-5 signaling exerts suppressive effects on these processes. The selection between these receptors is determined by the concentration of TGF-β, with ALK-1 preferentially activated at higher concentrations, thereby initiating signaling *via* Smad1, 5, and 8 [46].

Endothelial cell proliferation and migration regulation, along with the modulation of extracellular matrix synthesis, are critical roles attributed to TGF-β. It demonstrates a dose-dependent effect on angiogenesis, promoting migration and proliferation at low concentrations but inhibiting these processes at higher concentrations. This dual role suggests a possible mechanism for attracting and retaining cells during vascular repair [17]. During vascular inflammation, the initial step in atherogenesis involves monocyte adherence to the endothelium *via* selectins, allowing leukocytes to roll and attach to VCAM-1 and ICAM-1. TGF-β inhibits the expression of adhesion proteins and chemoattractants like P-selectin, E-selectin, ICAM-1, IL-6, and IL-8, and regulates endothelial cell proliferation, migration, and apoptosis. Reduced TGF-β levels, combined with dietary fat, promote vascular endothelial activation and lipid lesion formation, while TGF-β administration mitigates acute endothelial dysfunction and neutrophil recruitment in animal models of reperfusion injury [48]. Endothelial dysfunction in atherosclerosis involves the modulation of the endothelial cell phenotype, characterized by “Type II Activation,” where transcription factors like NFκB induce the expression of VCAM-1, procoagulant molecules, and chemokines, facilitating leukocyte recruitment and inflammation. Disturbed flow patterns in lesion-prone areas exacerbate endothelial dysfunction, with genes like VCAM-1, tissue factor, IL-8, and MCP-1 playing key roles in disease progression [49].

Nitric oxide (NO), a critical mediator produced by endothelial nitric oxide synthase (eNOS), maintains vascular homeostasis by promoting vasodilation, inhibiting platelet aggregation, and preventing leukocyte adhesion. Physiological shear stress enhances NO production through the activation of TGF-β3 signaling, which subsequently induces Krüppel-like factor 2 (KLF2) and eNOS, thereby stabilizing endothelial cells and protecting against atherosclerosis. Interestingly, TGF-β3, rather than TGF-β1, plays a key role in this protective response, indicating isoform-specific effects in endothelial cells. Disruptions in this signaling pathway contribute to endothelial dysfunction, one of the earliest events in atherogenesis [50].

The MAPK pathway further integrates into TGF-β signaling, influencing endothelial cell function and immune responses. Research by Su *et al.* demonstrated that MEKK2 and MEKK3—upstream regulators of MAPK interact with TGF-β signaling to modulate the stability of endothelial cells. This interaction regulates vascular integrity by controlling endothelial cell proliferation, migration, and survival. Importantly, the cross-talk between MAPK and TGF-β signals impacts immune responses in endothelial cells, suggesting that these pathways not only regulate vascular homeostasis but also control inflammatory responses in atherosclerosis [51]. Furthermore, noncanonical TGF-β signaling through Smad7 modulates immune pathways in endothelial cells. Yi Yu from Xin-Hua Feng’s group highlighted that Smad7, beyond its traditional inhibitory role in the TGF-β pathway, interacts with STAT3 to influence immune responses. PRMT5-mediated methylation of Smad7 strengthens this interaction, promoting endothelial cell survival and anti-inflammatory responses. The cooperation between TGF-β and MAPK pathways, particularly through Smad7 regulation, underscores the importance of noncanonical signaling in maintaining endothelial homeostasis and preventing vascular inflammation. This integration of TGF-β, MAPK, and NO pathways highlights a complex regulatory network that maintains vascular integrity, and disturbances in this cross-talk can accelerate atherosclerosis progression and vascular remodeling [52, 53].

## 5 TGF-Β AND SMOOTH MUSCLE CELL DYNAMICS IN ATHEROSCLEROSIS

Smooth muscle cells (SMCs) are integral to arterial structure, constituting the majority of the tunica media in healthy arteries, while myofibroblasts, expressing smooth muscle α-actin isoforms, are prevalent in atherosclerotic lesions [54]. TGF-β signaling plays a crucial role in the differentiation of pluripotent cells into the smooth muscle lineage. While in adult human SMC, TGF-β inhibits migration and proliferation, its effects on rat aortic SMC and chick embryonic cardiac neural crest cells vary, stimulating mitogen production and growth in some contexts [55]. Moreover, TGF-β significantly influences the phenotype of human SMC by modulating cytoskeletal organization and cell spreading. The impact of TGF-β on apoptosis is complex and context-dependent, with varying effects on different cell types. While it can induce apoptosis in vascular SMC and endothelial cells under certain conditions, it may promote cell survival in others. Lesion-derived cells, once established in culture, often display resistance to TGF-β-induced apoptosis, suggesting a role in wound or lesion regression post-vascular repair [17].

During atherosclerosis, TGF-β exhibits a dual regulatory role in the modulation of SMCs. While it typically inhibits SMC activation, proliferation, and migration, its effects can vary depending on concentration and context. High levels of TGF-β maintain SMCs in a contractile and differentiated state, contributing to vessel wall stability. However, low concentrations of TGF-β, as seen in conditions like unstable angina, may promote SMC proliferation and migration, exacerbating lesion formation [25]. Moreover, TGF-β1 expression is elevated in experimental models of vascular injury and human restenosis lesions, indicating its involvement in neointimal hyperplasia. Therefore, TGF-β emerges as a crucial regulator of SMC dynamics in atherosclerosis, influencing both protective and pathogenic processes [48]. SMCs primarily regulate pulse pressure and blood flow through contraction postnatally. They can adapt their phenotype, transitioning to proliferative, matrix synthetic cells in response to vascular injury. TGF-β governs both embryonic SMC differentiation and postnatal phenotypic switching. Its overexpression increases neointimal formation, smooth muscle proliferation, and differentiation in balloon injury models, suggesting its role in re-differentiation. TGF-β involvement spans various cardiovascular diseases, including atherosclerosis, congenital heart diseases, aortic aneurysm, hypertension, and hereditary hemorrhagic telangiectasia, often stemming from dysregulated SMC function or differentiation [56].

TCE, a crucial cis-element, regulates SMC marker genes such as α-SMA and SM22α. Mutations in TCE disrupt TGF-β-induced promoter activity, underlining its significance. Interactions with Kruppel-like transcription factors KLF4 and KLF5 further complicate this regulation, with KLF4 repressing TCE activity while KLF5 enhances it. TGF-β, by inducing miR-143 and miR-145, inhibits KLF4 expression, adding another layer to the intricate modulation of SMC marker gene expression [57]. CARg elements, present in SMC-specific gene promoters, play a pivotal role in TGF-β-induced SMC marker gene expression by binding to SRF. SRF overexpression enhances SMC marker expression and induces morphological changes [58]. Myocardin, a transcriptional cofactor highly expressed in aortic medial SMCs, is indispensable for SMC differentiation, promoting a contractile phenotype and SMC marker transcription. Although myocardin is considered a master regulator, its role in the initiation of SMC differentiation is debated, with some progenitor cells requiring a threshold level of myocardin for differentiation [59]. *in vivo* studies suggest myocardin's dispensability for vascular SMC development in certain contexts, while *in vitro* data propose Smad3's involvement in the initiation of TGF-β-induced SMC differentiation, with myocardin contributing to later-stage maturation [60]. Moreover, Smads serve as key mediators of TGF-β signaling, activating gene transcription by binding to SBEs in SMC marker gene promoters. Smad3, particularly, activates SBEs in the SM22α promoter, which is essential for SMC differentiation. Alongside Smads, RhoA and its downstream effector ROCK regulate SMC contractile function and marker gene expression [55]. RhoA activation induces a contractile phenotype, while its inhibition disrupts SMC differentiation by impairing Smad signaling, highlighting its importance in TGF-β-mediated SMC differentiation [61]. Among the multitude of pathways implicated in SMC regulation, the TGF-β/Smad signaling pathway stands out as paramount in governing the initiation of SMC differentiation. Notably, aberrations in this pathway appear to hold particular significance in the pathogenesis of atherosclerosis, suggesting its pivotal role compared to other regulatory routes in this disease process [62].

Eicosanoids also play a pivotal role in SMC behavior during the pathogenesis of atheroma through their involvement in chronic inflammation, vascular remodeling, and the regulation of endothelial and smooth muscle function. Pro-inflammatory prostanoids, such as prostaglandins and thromboxane A2 (TxA2), contribute to plaque formation by promoting endothelial dysfunction, vascular smooth muscle cell (VSMC) migration, and immune cell recruitment. In particular, TxA2 has been shown to enhance SMC proliferation and migration while promoting vasoconstriction and platelet aggregation, thereby accelerating atherogenesis [63]. Moreover, interactions between eicosanoids and TGF-β signaling pathways exacerbate vascular pathology by modulating SMC phenotypic switching. TGF-β, a key modulator of fibrosis and immune regulation, promotes collagen deposition and extracellular matrix remodeling in atherosclerotic plaques, reinforcing the fibrotic response. Pro-inflammatory eicosanoids such as PGE2 and PGF2α can upregulate TGF-β expression in macrophages and VSMCs, creating a feedback loop that sustains chronic inflammation and drives plaque progression. Conversely, anti-inflammatory eicosanoids like prostacyclin (PGI2) and epoxyeicosatrienoic acids (EETs) mitigate TGF-β-induced fibrotic responses in SMCs, protecting against excessive plaque development and maintaining vascular homeostasis. Thus, the dynamic interplay between pro-inflammatory and anti-inflammatory eicosanoids, in conjunction with TGF-β signaling, critically influences SMC behavior, determining the progression and stability of atheromatous plaques [63].

## 6 EXTRACELLULAR MATRIX REMODELING: TGF-Β AS A MODULATOR OF ATHEROSCLEROTIC PLAQUE STABILITY

The extracellular matrix (ECM), an intricate network abundant in polysaccharide modifications, is crucial in modulating the activity and availability of growth factors. By binding to these factors, the ECM limits their diffusion and access to receptors, acting as a reservoir that establishes necessary concentration gradients for cellular differentiation [64]. TGF-β is a versatile cytokine that controls the expression of ECM-associated genes during initial injury phases. The activity of TGF-β is greatly affected by substrate stiffness, with lower stiffness substrates hindering TGF-β protein activation [65]. Moreover, the ECM aids in the maturation of ligands by storing TGF-β in an inactive form until it is activated by matrix metalloproteinases (MMPs) or mechanical stress. The selective binding capacity of the ECM is essential for determining ligand-receptor specificity and guiding epithelial-mesenchymal signaling [66].

Atherosclerosis stems from lipoprotein accumulation in artery walls, prompting inflammation and repair mechanisms. The balance between these responses determines plaque stability: stable plaques have thick fibrous caps with low inflammation and lipids, while high-risk plaques are characterized by thin caps, reduced ECM, inflammation, and lipid accumulation. Hence, uncovering pathways for vascular repair and plaque stability is crucial for developing effective therapies. The TGF-β signaling pathway is a promising candidate, known for its anti-atherogenic effects. TGF-β isoforms (-β1, -β2, and -β3) are key regulators of cellular processes like proliferation, differentiation, tissue repair, ECM synthesis, and inflammation modulation [38].

TGFβ plays a pivotal role in various biological processes such as cell proliferation, migration, and extracellular matrix production. Its involvement in atherosclerotic plaques is well-established, with expressions of TGFβ ligands, receptors, and Smad proteins documented. However, its role in atherosclerosis remains contentious, with conflicting reports of both pro- and anti-atherosclerotic effects. Apart from its known anti-inflammatory properties, inhibiting endothelial cell TGFβ signaling enhances the expression of genes regulating vascular permeability, thereby reducing the typically heightened permeability associated with inflammation and atherosclerosis [7]. TGF-β serves as a pivotal regulator of ECM accumulation and is implicated in fibrosis. It stimulates the production of ECM components through its isoforms [67]. It promotes ECM synthesis, particularly collagen types I, III, IV, and V, fostering plaque stability. Conversely, pro-inflammatory cytokines like interleukin-1 (IL-1), tumor necrosis factor-alpha (TNF-α), and interferon-gamma (IFN-γ) suppress collagen production, exacerbating plaque instability [68].

TGF-β is widely recognized for its robust induction of inflammation, fibrosis, and activation of select MMPs. Moreover, TGF-β signaling begins with TGF-β binding to its receptors, triggering downstream signaling cascades through the release of mature TGF-β, which is regulated by proteases or MMPs [69]. Precisely, MMP-2, MMP-3, MMP-7, and MMP-9 play a pivotal role in liberating TGFβ from the ECM. An important function of MMPs is to break down growth factors and cytokines bound to the ECM, thereby compromising plaque stability, including that of TGF-β. The dynamic interaction among TGF-β, MMPs, and ECM turnover underscores their pivotal roles in atherosclerotic plaque stability and rupture susceptibility [70]. Furthermore, within the ECM, proteins such as TIMPs, which stand for Tissue Inhibitors of MMPs, are essential for counteracting the proteolytic activity of MMPs and preserving ECM integrity. TIMP-1 stands out as the most reliable indicator of TGF-β1 levels, a distinctive feature of fibrotic processes [71].

Table **1** highlights the key genes and proteins induced by TGF-β and their roles in endothelial dysfunction, VSMCs dynamics, and ECM remodeling in the context of atherosclerosis.

## 7 EMERGING TECHNOLOGIES IN CARDIOVASCULAR DISEASES

The landscape of cardiovascular disease treatment has evolved significantly with the advent of gene-editing technologies and RNA-based therapies, which hold the potential to address the underlying genetic causes of atherosclerosis and other cardiovascular disorders [72]. Traditional approaches to cardiovascular diseases primarily focus on managing symptoms through pharmacological interventions, but recent advancements in CRISPR/Cas9 and RNA interference (RNAi) offer curative potential by directly targeting pathogenic genes and molecular pathways involved in disease progression [73].

### 7.1 CRISPR/Cas9 in Cardiovascular Diseases

CRISPR/Cas9 technology represents one of the most revolutionary advancements in the field of genetic engineering, offering unprecedented precision in genome editing with applications across numerous diseases, including CVDs [74]. At the core of this technology is a two-component system comprising the Cas9 protein and a guide RNA (gRNA). The Cas9 protein functions as a nuclease that induces double-strand breaks (DSBs) at targeted genomic sites, while the gRNA directs Cas9 to the specific DNA sequence to be edited [75, 76]. This programmable system enables precise gene modifications by leveraging the cell’s natural DNA repair mechanisms, primarily non-homologous end joining (NHEJ) or homology-directed repair (HDR). The precision of this technology lies in the ability to design the gRNA to target specific DNA sequences, reducing the likelihood of off-target effects. However, ensuring high specificity and minimizing unintended edits remains a challenge in clinical applications [77, 78].

In the context of atherosclerosis, CRISPR-Cas9 has shown immense promise in targeting genes involved in lipid metabolism and inflammation, which are critical pathways in the development of atherosclerotic plaques [79]. Atherosclerosis is a chronic inflammatory condition characterized by the accumulation of lipids and immune cells within the arterial walls, leading to plaque formation and increased risk of cardiovascular events. One of the primary genetic targets in atherosclerosis research has been the PCSK9 gene, which encodes a protein involved in the degradation of low-density lipoprotein receptors (LDLR) [80]. Elevated levels of PCSK9 result in reduced LDLR availability, leading to higher circulating LDL cholesterol levels—a major risk factor for atherosclerosis. By using CRISPR-Cas9 to disrupt the PCSK9 gene, researchers have demonstrated significant reductions in plasma cholesterol levels and a decrease in atherosclerotic plaque formation in preclinical models. For example, *in vivo* studies using adenoviral vectors to deliver CRISPR components targeting PCSK9 have shown a 35-40% reduction in plasma cholesterol levels without notable off-target effects [81, 82].

Despite the promising results in preclinical studies, the delivery of CRISPR-Cas9 components to target tissues remains a significant challenge in cardiovascular applications. Efficient and tissue-specific delivery systems are essential for translating genome editing technologies into clinical therapies. Several delivery systems have been explored, each with distinct advantages and limitations. Adeno-associated viral (AAV) vectors are commonly used for delivering CRISPR components due to their ability to achieve long-term expression of the editing machinery [82]. However, AAV vectors have limited packaging capacity and can elicit immune responses, which may pose risks in clinical settings. Dual AAV systems have been developed to address the size limitation by splitting the CRISPR components into two separate vectors. However, this approach requires co-infection of the same cell, reducing overall efficiency [83]. Lipid nanoparticles (LNPs) offer a non-viral alternative for delivering CRISPR components, particularly mRNA encoding Cas9 and sgRNA. LNPs have shown promise in liver-targeted applications due to their low immunogenicity and ability to protect the cargo from degradation. However, their efficiency in targeting cardiovascular tissues, such as the heart and arterial walls, remains limited. Researchers are actively exploring strategies to enhance the specificity and efficiency of LNPs for cardiovascular applications [84, 85]. Another delivery approach involves direct microinjection of CRISPR components into target tissues or embryos. This method has been used to correct genetic mutations in preclinical models of hypertrophic cardiomyopathy and atherosclerosis, demonstrating the feasibility of *in vivo* genome editing. However, microinjection is not practical for widespread clinical use due to its invasive nature and the difficulty of targeting specific tissues [86].

Mechanistically, CRISPR-Cas9 can influence atherosclerosis by modulating key pathways involved in lipid metabolism, inflammation, and vascular repair. For example, the disruption of PCSK9 and APOC3 genes directly impacts lipid profiles, reducing the availability of atherogenic lipoproteins in the bloodstream [87, 88]. This reduction in circulating LDL cholesterol and triglycerides can slow the progression of plaque formation and reduce the risk of plaque rupture—a major cause of myocardial infarction and stroke [89]. In addition to lipid modulation, CRISPR-Cas9 can be used to target pro-inflammatory cytokines and signaling pathways, thereby mitigating the chronic inflammation that drives atherosclerotic progression. Targeting genes such as IL6, TNF, and NF-κB could reduce the recruitment of immune cells to the arterial wall and decrease the secretion of pro-inflammatory mediators, ultimately stabilizing plaques and reducing the risk of acute cardiovascular events [90].

### 7.2 RNA-Based Therapies in Cardiovascular Diseases

RNA-based therapies represent a promising frontier in the treatment of cardiovascular diseases (CVDs), offering novel approaches to target previously inaccessible pathways. RNA therapeutics, including messenger RNAs (mRNAs), antisense oligonucleotides (ASOs), small interfering RNAs (siRNAs), and microRNAs (miRNAs), offer precise control over gene expression. These therapies function by either silencing disease-promoting genes or introducing exogenous RNA to replace deficient proteins. The transformative potential of RNA-based treatments, demonstrated by the success of COVID-19 mRNA vaccines, has catalyzed their development in cardiovascular research. Unlike traditional small molecules, RNA therapeutics can be designed to target proteins with complex conformations or non-enzymatic functions that are inaccessible to conventional drugs [91].

RNA-based therapeutics address various cardiovascular conditions by modulating gene expression. For instance, antisense oligonucleotides can bind to specific mRNA sequences, preventing their translation and thereby reducing the production of disease-related proteins [92]. In cardiovascular diseases, ASOs have shown promise in managing hypercholesterolemia by targeting apolipoprotein B-100 mRNA. Another approach involves the use of siRNAs, which harness the RNA-induced silencing complex (RISC) to degrade target mRNA. Inclisiran, a siRNA drug targeting PCSK9 mRNA, effectively reduces LDL cholesterol levels and is currently in clinical use [93-95].

MicroRNAs, a subclass of non-coding RNAs, also play a critical role in cardiovascular health by regulating multiple gene networks simultaneously. miRNA-based therapies have shown potential in promoting cardiac regeneration and mitigating fibrosis. The inhibition of miR-132, for instance, has demonstrated efficacy in reducing heart failure progression [96]. Additionally, synthetic mRNAs can be used to produce therapeutic proteins directly within target cells. An example is the VEGF-A mRNA therapy, which aims to stimulate angiogenesis in ischemic heart tissues, thereby improving perfusion and myocardial recovery [97].

RNA-based therapies for cardiovascular diseases are progressing rapidly, with several candidates in clinical trials. One of the earliest RNA therapies was Mipomersen, targeting apolipoprotein B-100 mRNA to treat hypercholesterolemia. Although discontinued due to hepatotoxicity, it paved the way for safer second-generation ASOs, such as TQJ230 and volanesorsen, which have shown significant efficacy in lowering lipoprotein levels [98, 99]. Inclisiran, a siRNA drug targeting PCSK9, has demonstrated long-term cholesterol reduction with biannual dosing, making it a promising alternative to monoclonal antibody therapies [95].

## 8 GENETIC APPROACHES TARGETING TGF-β SIGNALING FOR CARDIOVASCULAR DISEASES

### 8.1 LXRα

Initial approaches to treating atherosclerosis relied on gene editing to modify lipid metabolism. Atherosclerosis involves the accumulation of lipids, particularly low-density lipoprotein (LDL), in the arterial walls. Researchers have been exploring CRISPR-based gene editing to modify genes involved in lipid metabolism to reduce LDL levels or enhance high-density lipoprotein (HDL) levels, which could potentially mitigate atherosclerosis progression. TGF-β1 shows potential in the treatment of atherosclerosis by regulating cholesterol metabolism in macrophage-derived foam cells. TGF-β1 significantly increases the expression of key cholesterol transporters, including ABCA1, ABCG1, and SR-BI, at both transcriptional and translational levels. This upregulation enhances cholesterol efflux from foam cells, thereby reducing their cholesterol content. Moreover, TGF-β1 activates Liver X Receptor Alpha (LXRα), a critical regulator of cholesterol homeostasis. The promotion of cholesterol efflux by TGF-β1 is dependent on the activation of LXRα, as evidenced by the fact that silencing LXRα with small interfering RNA negates TGF-β1's effects [100]. In a related study, Li *et al.* transfected hematopoietic stem cells (HSCs) with lentivectors-loaded LXRα and transplanted these HSCs into an LDLR−/− mouse model of atherosclerosis. This intervention resulted in a reduction of both plasma triglyceride levels and atherosclerotic plaque volume. The protective effect on atherosclerosis is attributed to the enhanced expression of cholesterol efflux genes ABCA1 and apoE by LXRα. Additionally, LXRs are involved in regulating cytokine production, such as interleukin 1 beta (IL-1β), interleukin 6 (IL-6), and tumor necrosis factor-α (TNF-α). The study observed that IL-6 and TNF-α levels were down-regulated when the transgene enhanced LXRα. Therefore, the authors suggest that transgenic LXRα helps inhibit the progression of atherosclerosis by regulating both lipid metabolism and the inflammatory response [5].

### 8.2 KLF11

Currently, targeting inflammation has become a trend in cardiovascular medicine. Gene modulation can be utilized to regulate the expression of inflammatory mediators or target immune cells involved in the inflammatory response within the arterial walls. For instance, modifying endothelial cells or smooth muscle cells to enhance their protective functions or reduce their pro-atherogenic properties could be a potential strategy. Krüppel Like Factor 11 (KLF11), also known as TGF-β inducible immediate early gene (TIEG), has emerged as a noteworthy target [101]. The inflammatory status of endothelial cells (ECs) plays a crucial role in many vascular diseases. However, its role in the cardiovascular system is not well understood. Researchers, led by Yanbo Fan, sought to explore KLF11's role in vascular endothelial inflammation [102]. KLF11 is highly expressed in ECs and is upregulated by pro-inflammatory stimuli. Overexpression of KLF11 *via* adenovirus reduces the expression of tumor necrosis factor (TNF)-α-induced adhesion molecules. Conversely, knocking down KLF11 with siRNA increases the pro-inflammatory status of ECs. KLF11 suppresses the promoter activity of adhesion molecules induced by TNF-α and NF-κB p65 overexpression. It achieves this by physically interacting with p65, thereby inhibiting the NF-κB signaling pathway. KLF11 knocked down results in an increased binding of p65 to VCAM-1 and E-selectin promoters. *in vivo*, KLF11-/- mice show a significant rise in leukocyte recruitment to ECs following lipopolysaccharide (LPS) administration. These findings reveal that KLF11 acts as a suppressor of EC inflammatory activation, making it a novel potential target for treating vascular inflammatory diseases [102].

### 8.3 Smad2/3

To specifically investigate the role of Smad2/3 signaling in the endothelium, researchers used Tie2-Cre transgenic mice to delete the Smad2 gene in Smad3 knockout mice, creating EC-specific Smad2/3 double knockout (EC-Smad2/3KO) mice. These embryos exhibited hemorrhage and embryonic lethality around E12.5 While vasculogenesis and angiogenesis appeared normal in the yolk sac and the whole embryo, the EC-Smad2/3KO embryos showed incomplete vascular maturation due to insufficient assembly of mural cells. The vasculature of EC-Smad2/3KO mice had wide gaps between ECs and mural cells, linked to reduced expression of N-cadherin and sphingosine-1-phosphate receptor-1 (S1PR1) in these ECs. These findings indicate that Smad2/3 signaling in ECs is crucial for maintaining vascular integrity by regulating the expression of N-cadherin, VE-cadherin, and S1PR1. Aberrant vascularization leads to various diseases, including atherosclerosis. This research highlights Smad2/3 as a potential therapeutic target, as modulating this pathway could enhance vascular integrity and maturation, offering new avenues for treating vascular diseases [103]. The role of Smad3 in vascular inflammation was previously described by Feinberg *et al.* Expanding upon their findings, this study delves deeper into the modulation of TGFβ1 and its impact on vascular inflammation, a field ripe for exploration. Monocyte chemoattractant protein (MCP)-1, critical in macrophage recruitment during inflammation, emerges as a focal point. Through meticulous investigation, this research definitively identifies Smad3 as the pivotal mediator of TGF-β1's suppression of MCP-1 expression. Demonstrating the efficacy of Smad3 overexpression in stifling MCP-1 expression, while Smad3 deficiency in macrophages abolishes TGF-β1's inhibition of MCP-1, underscores the significance of Smad3 in this regulatory process. As a consequence of this deficiency, accelerated intimal hyperplasia and increased infiltration of MCP-1-expressing macrophages in cardiac allografts were observed. Moreover, a novel mechanism is unveiled wherein Smad3 antagonizes AP-1 DNA-protein binding and activity, shedding new light on its regulatory role. Consequently, targeting Smad3 emerges as a promising avenue for mitigating vascular inflammation in conditions such as transplant-associated arteriopathy, thus pesenting itself as a potential therapeutic target [104]. The recent research findings, led by Deng *et al.*, elucidated that Smad2/3 plays a pivotal role in endothelial cell (EC) sensing of wall fluid shear stress (FSS) and subsequent vessel remodeling. These results shed light on the intricate mechanisms underlying FSS-induced vascular responses, particularly the signaling cascade involving the Alk5 receptor and Neuropilin-1, which induce Smad2/3 phosphorylation. The observed maximal Smad2/3 nuclear translocation and target gene expression at low FSS underscore its significant involvement in inward artery remodeling. Therefore, these recent findings underscore the therapeutic potential of targeting Smad2/3 in managing conditions characterized by aberrant vessel remodeling, such as atherosclerosis [105].

### 8.4 ALK-1/Smad1

Under María González-Núñez's leadership, researchers have identified ALK-1/Smad1 as a promising therapy target, linking it to various physiological and pathological processes. ALK-1's role in cardiovascular homeostasis extends beyond endothelial biology to vascular smooth muscle cells and inflammatory cells, potentially affecting arterial pressure regulation and inflammatory cell infiltration. Although the specifics of ALK-1's involvement in fibrotic and remodeling processes remain less explored, they could contribute to significant cardiovascular pathologies. The pleiotropic effects of TGF-β across different cell types, including endothelial cells, vascular smooth muscle cells, monocytes, macrophages, and fibroblasts, highlight the complexity of ALK-1's functions. Understanding how this signaling pathway regulates vascular homeostasis could lead to novel therapeutic strategies involving its activation or inhibition. Certain ALK-1 inhibitors, like PF-3446962 and ACE-041, have shown promise in cancer therapy by blocking BMP-9/BMP-10 signaling through ALK-1, inhibiting sprout formation. Additionally, the inhibition of TGF-β signaling by BAMBI (BMP and Activin receptor Membrane Bound Inhibitor) presents a potential strategy for modulating cardiovascular remodeling and angiogenesis. Uncovering ALK-1's functions and therapeutic implications offers new possibilities in cardiovascular medicine, potentially improving treatments for cardiovascular diseases [106].

### 8.5 Tgfbr1/2

**Fig. (1):**
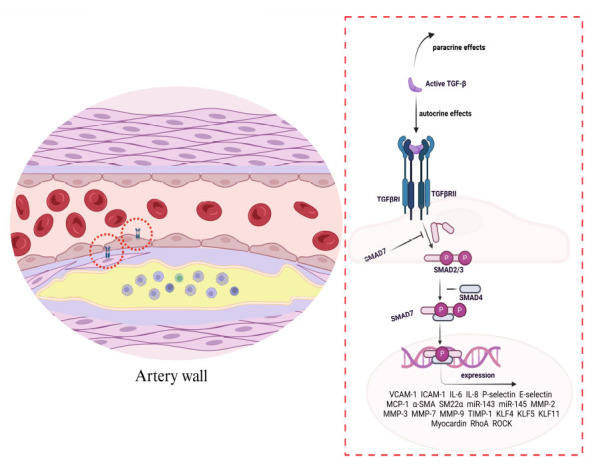
TGF-β signaling pathway plays a central role in regulating vascular cell behavior, particularly in endothelial cells and vascular smooth muscle cells (VSMCs). Activated TGF-β binds to its receptors (TGFBRI and TGFBRII), initiating a cascade that phosphorylates SMAD2/3. The SMAD2/3 complex associates with SMAD4, translocating to the nucleus to regulate gene expression. Key TGF-β-regulated genes include those involved in endothelial dysfunction, inflammation, smooth muscle differentiation, and extracellular matrix remodeling. Created by Biorender 2024.

Based on research led by Chen *et al.*, a potential therapeutic avenue for reducing inflammation in atherosclerosis has been explored by targeting the transforming growth factor-beta receptors (TGFβRs). In their study, they investigated the effects of deleting TGFβ receptors, specifically in ECs, using mouse models. The study revealed that mice with TGFβ receptor deletion in ECs exhibited delayed onset and reduced extent of lipid deposition in their blood vessels when subjected to a high-cholesterol, high-fat diet. This reduction in lipid buildup was substantial, ranging from 61% to 79% compared to control mice over the study period. These findings suggest that blocking TGFβ signaling, specifically in ECs could represent an effective strategy for slowing down the progression of atherosclerosis (Fig. **1**) [7].

## 9 FUTURE PERSPECTIVES IN TGF-Β SIGNALING MODULATION

### 9.1 Limitations and Future Directions in Targeting TGF-β Signaling

Gene therapy has revolutionized the field of genetic medicine by enabling the precise modification of genetic material to treat a wide range of diseases. One of the most promising applications of gene therapy is in the treatment of atherosclerosis, a major cardiovascular disease characterized by the buildup of plaque in the arterial walls. By targeting the underlying genetic causes and pathways involved in cholesterol metabolism and inflammation, gene therapy offers a potential cure rather than merely managing symptoms [107]. The CRISPR/Cas9 technology stands out as a prominent example of gene therapy due to its ability to make precise, targeted modifications to the genome. In the realm of cardiovascular disease therapy, CRISPR technology shows significant potential, particularly in harmonizing cell functions at the target site by targeting specific genes involved in cholesterol metabolism [108]. Inactivating genes such as PCSK9, APOC3, and ANGPTL3 has demonstrated athero-protective effects. For example, CRISPR-mediated inhibition of PCSK9 in mice reduced plasma cholesterol by 30-40%. Similar approaches in humanized mouse models also showed decreased human PCSK9 levels. Studies using human induced pluripotent stem cells (hiPSCs) have demonstrated the significant role of PCSK9 in modulating TGFβ-NODAL signaling, impacting key processes in cell biology [109]. In the context of TGF-β signaling, CRISPR can be employed to both activate and inhibit this critical pathway, which plays a key role in processes like cell growth, differentiation, and immune responses. By targeting specific genes within the TGF-β signaling cascade, it is possible to manipulate this pathway to study its functions across various biological contexts. Recent investigations have delved into the manipulation of transcriptional co-repressors and co-activators as a means to modulate TGF-β signaling and its downstream ramifications, particularly in the context of regulating fibrosis-associated genes such as alpha-smooth muscle actin (α-SMA). In a notable study focusing on corneal wound healing, CRISPR/Cas9 technology was harnessed to edit the TGIF1 gene, a transcriptional repressor intricately involved in α-SMA expression regulation. Through the activation of TGIF1, researchers observed a noteworthy decrease in myofibroblast formation and the expression of profibrotic genes upon TGF-β1 stimulation in primary human corneal stromal fibroblasts. This investigation underscores the potential of CRISPR-based approaches in elucidating the intricate mechanisms of TGF-β signaling, particularly the TGFβ1/Smad pathway while suggesting novel therapeutic avenues for addressing fibrosis-related pathologies [110]. Despite the promise of gene therapy, including CRISPR/Cas9, several limitations must be addressed. Commonly used gene delivery methods, particularly viral vectors, are associated with toxicity and immunological reactions. These adverse effects pose significant challenges for safe and effective gene therapy applications. Additionally, the small packaging capacity of certain viral vectors, such as adeno-associated viruses (AAV), restricts their use for delivering larger genetic payloads [111, 112]. Non-viral vectors are being explored as alternatives to mitigate these risks, but they come with their challenges. The increased prevalence of cardiovascular diseases (CVD) and the high costs associated with gene therapy further complicate its widespread adoption. Moreover, the complex pathophysiology of CVD and the incomplete understanding of the mechanisms driving these diseases pose significant obstacles to the broad implementation of gene therapy in clinical practice. Adverse events, such as off-target effects and immunotoxicity, have been reported in CRISPR/Cas9 applications, highlighting the need for further research and refinement of delivery systems [113].

### 9.2 Personalized Strategies in TGF-β-Targeted Therapies

Personalized approaches to atherosclerosis aim to shift the focus from managing advanced cardiovascular events to early detection and targeted prevention strategies tailored to an individual’s unique risk profile [114]. Given the genetic heterogeneity among patients, a one-size-fits-all approach to TGF-β modulation is insufficient to address the complex mechanisms underlying cardiovascular diseases such as atherosclerosis and arterial stiffness.

Single nucleotide polymorphisms (SNPs) represent key genetic variations that can influence the susceptibility and progression of various diseases, including cardiovascular conditions. Polymorphisms in the TGF-β1 gene, such as C509T (rs1800469) and T869C (rs1800470), have been shown to impact the risk and prognosis of ischemic heart disease (IHD), particularly in specific populations like the Iraqi cohort. These genetic variations can alter TGF-β1 expression levels, affecting cardiovascular outcomes. Studies have identified protective haplotypes, such as H4 (T509-C869), that may reduce IHD risk, suggesting that genetic screening for these variants could help refine cardiovascular risk stratification. Elevated TGF-β1 levels are associated with pathological changes such as myocardial fibrosis and atherosclerosis, underscoring the importance of SNP analysis in identifying individuals with a higher genetic predisposition to cardiovascular diseases [115]. It is important to note that the examples of SNPs provided are part of a much broader landscape of genetic variations within the TGF-β pathway. Numerous other SNPs in different populations likely contribute to cardiovascular risk, although not all of them have been extensively studied. The ongoing identification of additional polymorphisms could refine our understanding of how TGF-β signaling impacts cardiovascular diseases. Genetic studies focusing on different cohorts, ethnicities, and disease subtypes continue to uncover novel variants, further supporting the importance of personalized approaches in cardiovascular medicine.

Building upon the genetic basis of cardiovascular disease, the TGF-β pathway has garnered attention due to its pivotal role in regulating vascular remodeling and fibrosis. Beyond polymorphisms in TGF-β1, variations in other key genes within this signaling cascade, particularly TGFBR2, have been implicated in cardiovascular pathology. A study investigating patients in a Caucasian population with CAD identified the rs9838682 polymorphism in TGFBR2 as a genetic variant significantly associated with an increased risk of sudden cardiac arrest (SCA). This SNP lies in a region of high linkage disequilibrium, suggesting it may either directly influence gene function or be in proximity to a causative variant affecting TGF-β signaling. Alterations in TGF-β signaling contribute to maladaptive cardiac remodeling processes, including myocardial fibrosis, which increases the susceptibility to ventricular arrhythmias and ischemic events. These findings underscore the clinical relevance of incorporating TGFBR2 polymorphisms into genetic screening, as variations within this receptor gene may refine cardiovascular risk stratification beyond traditional biomarkers. Personalized approaches targeting TGF-β signaling could offer more precise preventive strategies, particularly in individuals with a genetic predisposition to adverse cardiac outcomes such as myocardial infarction or SCA. Emerging genomic technologies, including whole-genome sequencing, facilitate the identification of patients with specific genetic predispositions to TGF-β pathway dysregulation. Patients with loss-of-function mutations in TGFBR2, for example, might benefit more from therapies aimed at enhancing TGF-β signaling. In contrast, individuals with gain-of-function mutations may require inhibitory approaches to prevent excessive fibrosis and inflammation. The ability to stratify patients based on their genetic profiles enables the development of targeted therapies that maximize efficacy while minimizing adverse effects [116].

Further supporting the importance of genetic polymorphisms in TGF-β signaling pathways, a study conducted on the Chinese Han population assessed the association between TGF-β1 (T29C) and TGFBR2 (rs6785385 and rs764522) gene polymorphisms and CAD severity. While the TGFBR2 variants showed no significant correlation with CAD occurrence or severity, the T29C polymorphism in TGF-β1 was independently associated with more severe coronary stenosis, particularly in male patients. The study demonstrated a positive correlation between the T allele of the T29C polymorphism and the number of coronary arteries affected, as well as with the Gensini score—a measure of CAD severity. Interestingly, patients carrying the T allele presented with a higher risk of advanced atherosclerosis and increased coronary narrowing. This finding aligns with earlier research indicating that variations in TGF-β1 expression may influence the progression of atherosclerosis through altered extracellular matrix deposition and fibrotic remodeling. Collectively, these data emphasize the importance of personalized genetic screening for TGF-β-related polymorphisms to identify high-risk individuals and guide targeted therapeutic interventions, especially in populations where specific genetic variants may be prevalent [117].

Polymorphisms in downstream mediators of the TGF-β signaling pathway, such as SMAD3, are also associated with cardiovascular diseases. SMAD3 plays a key role in translating extracellular TGF-β signals into gene expression changes in vascular cells. In a Slovenian population, the rs17228212 polymorphism of SMAD3 was linked to advanced carotid atherosclerosis, with the T allele increasing both disease risk and SMAD3 expression in plaques. Elevated SMAD3 levels may drive smooth muscle cell plasticity, promoting a pro-inflammatory and pro-remodeling phenotype, which contributes to plaque progression [118]. However, these findings contrast with studies by Samani and Rayat, who found no association between the rs17228212 polymorphism and cardiovascular risk. The discrepancies may be explained by differences in sample size and population characteristics, particularly as Rayat *et al.* conducted their study on a smaller Iranian cohort and focused on coronary artery disease rather than carotid atherosclerosis. These variations highlight the complexity of genetic influences across different populations and cardiovascular conditions. These findings emphasize that genetic variations in TGF-β mediators influence vascular remodeling and highlight the importance of SMAD3 screening in improving personalized risk assessments for atherosclerotic diseases [119].

## CONCLUSION

Targeting and modulating TGF-β signaling through gene therapy hold great promise for revolutionizing the treatment of atherosclerosis. TGF-β plays a multifaceted role in atherosclerosis, influencing endothelial dysfunction, smooth muscle cell dynamics, and extracellular matrix remodeling. By targeting specific genes within the TGF-β/Smad pathway, we can potentially modulate key processes involved in disease progression, such as inflammation, cholesterol metabolism, and plaque stability. CRISPR/Cas9 technology provides a precise tool to correct dysregulated TGF-β pathways, opening the door to new therapeutic approaches. Overcoming the limitations of current gene therapy techniques will be essential to exploit these advances to effectively prevent and treat atherosclerosis fully. The future of atherosclerosis therapy lies in personalization. Genetic variability across individuals significantly impacts the efficacy of gene-based interventions, emphasizing the need to tailor treatment to patient-specific genetic profiles. Personalized approaches incorporating genomic data, such as SNP analysis in the TGF-β pathway, can improve the precision of therapeutic strategies. Technologies such as CRISPR/Cas9 and RNA-based therapies enable targeted modifications based on individual genetic predispositions, reducing the risk of adverse events while maximizing therapeutic efficacy. This precision medicine framework has the potential to transform cardiovascular care, moving from generic treatments to interventions designed to address the unique genetic drivers of disease in each patient. It lays the groundwork for *in vivo* or *ex vivo* applications aimed at treating various cell types through specialized strategies.

## AUTHORS’ CONTRIBUTIONS

The authors confirm their contributions to the papaer as follows: Conceptualization and writing—original draft preparation, K.B., D.J., K.L., J.L-B., I.W., M.G.; supervision, Y.B., M.G.; revision and approval of the final version of the manuscript, K.B., D.J., K.L., J.L-B., I.W., M.G.; Y.B., M.G. All authors have read and agreed to the published version of the manuscript.

## Figures and Tables

**Table 1:** Key mediators and players of the TGF-β and their functions.

**Gene/Protein**	**Role in Atherosclerosis and Endothelial Dysfunction**
**TGF-β Isoforms**	
TGF-β1	Regulates smooth muscle cell dynamics, contributing to neointimal hyperplasia. Promotes endothelial-to-mesenchymal transition and alters vascular permeability. Enhances cholesterol efflux by increasing ABCA1, ABCG1, and SR-BI expression in foam cells. Inhibits MCP-1 expression *via* Smad3 signaling, reducing macrophage recruitment. Released by platelets in coronary artery disease, contributing to atherothrombosis. Its activation by ROS links oxidative stress to vascular dysfunction. Plays both protective and pathogenic roles depending on the local cytokine environment.
TGF-β2	Enhances plaque stability by promoting collagen accumulation and inhibiting MMP-9, reducing the risk of plaque rupture. Acts as a key paracrine signal in EndMT, contributing to endothelial dysfunction. Regulates EndMT through both Smad-dependent and Smad-independent pathways. Induced by inflammatory cytokines like IL-1β, TNF-α, and IL-6, linking it to oxidative stress and vascular inflammation. Differentiates asymptomatic from symptomatic plaques, highlighting its role in plaque pathophysiology.
TGF-β3	Contributes to endothelial stability by enhancing NO production through activation of KLF2 and eNOS. Plays a key role in protecting endothelial cells from dysfunction under physiological shear stress. Facilitates wound healing processes but exhibits pleiotropic effects depending on the context. Disruptions in its signaling contribute to early endothelial dysfunction, a critical step in atherogenesis. Its role in inflammatory regulation within atherosclerotic tissues remains under investigation.
**TGF-β-Associated Molecules**	
VCAM-1	Facilitates leukocyte recruitment and inflammation during endothelial dysfunction.
ICAM-1	Involved in leukocyte adhesion to the endothelium, promoting inflammation.
IL-6	Pro-inflammatory cytokine that can exacerbate endothelial dysfunction.
IL-8	Chemokine that attracts neutrophils, contributing to inflammation.
P-selectin	Adhesion molecule involved in leukocyte rolling and adhesion.
E-selectin	Similar to P-selectin, it aids in leukocyte rolling and adhesion.
MCP-1	Chemokine that recruits monocytes to the site of inflammation.
α-SMA	Smooth muscle cell marker gene regulated by TGF-β in SMC differentiation.
SM22α	Another SMC marker gene regulated by TGF-β is crucial for SMC differentiation.
miR-143	MicroRNA induced by TGF-β inhibits KLF4 expression is involved in SMC marker gene expression.
miR-145	Similar to miR-143, it regulates KLF4 expression and SMC marker gene expression.
MMP-2	Matrix metalloproteinase plays a role in liberating TGF-β from the ECM, influencing plaque stability.
MMP-3	Involved in ECM turnover and TGF-β activation.
MMP-7	Involved in ECM remodeling and TGF-β activation.
MMP-9	Contributes to ECM degradation and TGF-β activation, affecting plaque stability.
TIMP-1	Tissue inhibitor of MMPs regulates ECM integrity and counteracts MMP activity, indicative of TGF-β1 levels.
KLF4	Transcription factor repressed by miR-143 and miR-145 in SMCs.
KLF5	Transcription factor that enhances TGF-β-induced SMC marker gene expression.
Myocardin	Transcriptional cofactor promoting SMC differentiation and contractile phenotype.
RhoA	Regulates SMC contractile function and marker gene expression.
ROCK	Downstream effector of RhoA, involved in SMC differentiation and contractile function.

## LIST OF ABBREVIATIONS

SBE: Smad-binding Elements
Smad: Small Mothers Against Decapentaplegic
TGFβ: Transforming Growth Factor Beta
GARP: Glycoprotein-A Repetitions Predominant Protein
